# Use of emergency care services by immigrants—a survey of walk-in patients who attended the Oslo Accident and Emergency Outpatient Clinic

**DOI:** 10.1186/s12873-015-0055-0

**Published:** 2015-10-07

**Authors:** Sven Eirik Ruud, Ruth Aga, Bård Natvig, Per Hjortdahl

**Affiliations:** Department of General Practice, Institute of Health and Society, University of Oslo, Oslo, Norway; Department of Emergency General Practice, City of Oslo Health Agency, Oslo, Norway; Section for Orthopaedic Emergency, Oslo University Hospital, Oslo, Norway

**Keywords:** Emergency care utilization, Immigrant, General practice, Health-seeking behaviour, Primary health care, Regular general practitioner

## Abstract

**Background:**

The Oslo Accident and Emergency Outpatient Clinic (OAEOC) experienced a 5–6 % annual increase in patient visits between 2005 and 2011, which was significantly higher than the 2–3 % annual increase among registered Oslo residents. This study explored immigrant walk-in patients’ use of both the general emergency and trauma clinics of the OAEOC and their concomitant use of regular general practitioners (RGPs) in Oslo.

**Methods:**

A cross-sectional survey of walk-in patients attending the OAEOC during 2 weeks in September 2009. We analysed demographic data, patients’ self-reported affiliation with the RGP scheme, self-reported number of OAEOC and RGP consultations during the preceding 12 months. The first approach used Poisson regression models to study visit frequency. The second approach compared the proportions of first- and second-generation immigrants and those from the four most frequently represented countries (Sweden, Pakistan, Somalia and Poland) among the patient population, with their respective proportions within the general Oslo population.

**Results:**

The analysis included 3864 patients: 1821 attended the Department of Emergency General Practice (“general emergency clinic”); 2043 attended the Section for Orthopaedic Emergency (“trauma clinic”). Both first- and second-generation immigrants reported a significantly higher OAEOC visit frequency compared with Norwegians. Norwegians, representing 73 % of the city population accounted for 65 % of OAEOC visits. In contrast, first- and second-generation immigrants made up 27 % of the city population but accounted for 35 % of OAEOC visits. This proportional increase in use was primarily observed in the general emergency clinic (42 % of visits). Their proportional use of the trauma clinic (29 %) was similar to their proportion in the city. Among first-generation immigrants only 71 % were affiliated with the RGP system, in contrast to 96 % of Norwegians. Similar finding were obtained when immigrants were grouped by nationality. Compared to Norwegians, immigrants from Sweden, Pakistan and Somalia reported using the OAEOC significantly more often. Immigrants from Sweden, Poland and Somalia were over-represented at both clinics. The least frequent RGP affiliation was among immigrants from Sweden (32 %) and Poland (65 %).

**Conclusions:**

In Norway, immigrant subgroups use emergency health care services in different ways. Understanding these patterns of health-seeking behaviour may be important when designing emergency health services.

**Electronic supplementary material:**

The online version of this article (doi:10.1186/s12873-015-0055-0) contains supplementary material, which is available to authorized users.

## Background

The Norwegian population has become increasingly multicultural. In 2010, the population of immigrants and Norwegians born to immigrant parents comprised 11 % of the total Norwegian population and 27 % of the population in the capital, Oslo. This demographic change has introduced several challenges to the health care system, including maintaining equity of access and handling new patterns of health care utilization.

According to annual statistics, the Oslo Accident and Emergency Outpatient Clinic (OAEOC) experienced an average 5–6 % annual increase in patient numbers between 2005 and 2011. This is significantly higher than the 2–3 % annual increase among registered Oslo residents [1]. A study in the capital of Denmark, Copenhagen, concluded that immigrants have a higher proportion of non-urgent emergency room visits, presumably due to barriers in access to primary care [2]. This increased use of emergency services by immigrants may reflect cultural differences related to health literacy, poor knowledge about the health care system, inability to make appointments by phone due to language limitations, difficulties accessing a regular general practitioner (RGP) and illegal immigrant status [2–5]. Surveys and registry-based studies in Norway, Spain, Italy, Denmark, Great Britain, Sweden and the USA have reported variable results regarding immigrants’ utilization of emergency health care services [4, 6–14].

In 2001, Norway established a list-based patient system through which most inhabitants are assigned an RGP. Only individuals who are registered with the Norwegian National Population Register are eligible for enrolment in the RGP system [15]. Asylum seekers, refugees and their children who have been assigned a temporary identification number can register with a RGP or use a general health care service organized by the municipal authorities. Immigrants with an intention to stay in Norway for at least six months and who have been allocated a residence permit can register with the RGP scheme after they have received a personal identification number. Patients who fall outside the RGP system include undocumented immigrants, rejected asylum seekers and short-term immigrants working in Norway. However, like all citizens, they have the right to receive emergency health care within the health care system.

Throughout most of Norway, RGPs handle patients’ primary emergency care needs, but the situation is usually more complex in cities. If individuals become acutely ill during the daytime in Oslo, they are expected to seek help from their RGP during regular hours (08:00–16:00, Monday–Friday). However, if their RGP is unavailable or if they are not assigned to a RGP, individuals frequently use the Department of Emergency General Practice (the DEGP, or general emergency clinic), which is part of the larger OAEOC, or one of Oslo’s few and smaller private emergency care facilities. Outside of regular RGP working hours, individuals are expected to go to the OAEOC for urgent medical care. For minor injuries and trauma, individuals are expected to by-pass their RGP, regardless of the time of day, and proceed directly to the Section for Orthopaedic Emergency (SOE, or trauma clinic) at the OAEOC. Major trauma cases and other emergencies are admitted directly to the Emergency Department at Oslo University Hospital by ambulance or medical referral.

In the present study, we explored how immigrants, immigrant subgroups and native Norwegians use Oslo’s major emergency walk-in clinic and their concomitant use of RGPs. We used two analytic approaches. First, we compared subgroups’ self-reported use of the OAEOC, their self-reported affiliation with the RGP patient system and their number of RGP visits during the preceding 12 months. Second, we compared the proportions of immigrants in the patient population to their respective proportional representation in the overall population of Oslo.

## Methods

### Setting and study design

Patients who attended the OAEOC during a 2-week period in September 2009 were surveyed. A 2-week period was chosen due to time restrictions imposed by the OAEOC management. The emergency clinic is located in the centre of Oslo. It is the only government-run emergency outpatient clinic service open on a 24-h basis and is the largest emergency outpatient clinic in the city. It is organized as two separate clinics located within the same building. The general emergency clinic is staffed by general practitioners and operated by the Municipality of Oslo. The trauma clinic is integrated within the Orthopaedic Department of Oslo University Hospital and treats injuries and other minor trauma cases. In 2009, the OAEOC handled about 180,500 patients: 82,000 emergency admissions to the general emergency clinic, 72,000 emergency admissions to the trauma clinic and 26,500 follow-up appointments at the trauma clinic.

Individuals in need of emergency health services either attend as walk-in patients or are brought in by ambulance, the police or an emergency outreach team. All walk-in patients enter the OAEOC through the same entrance. A health secretary directs them to either the trauma clinic or the general emergency clinic depending on their health care needs. At both clinics they are attended by a triage nurse.

Study patients were included irrespective of when they were seen. Patients who fulfilled the inclusion criteria were asked by the triage nurse to participate in the study by answering a 15-item questionnaire (see Additional file 1). The questionnaire included items related to their and their parents’ countries of birth, their age, gender, work status and use of health care services during the preceding 12 months. Some of the questions were based on a study by the National Centre for Emergency Primary Health Care and the Norwegian Knowledge Centre for the Health Services [16]; other questions were written specifically for this survey. The questionnaire and attached information sheets were available in seven languages: Norwegian, English, Polish, Somali, Sorani (Kurdish), Farsi (Persian) and Urdu so that participants were able to select their preferred language version. Translators from the Municipal Interpreting and Translation Service of Oslo were consulted regarding which languages to include and prepared the translations. Each language version was examined and proofread by an independent translator who compared it with the original Norwegian text. Inconsistencies were resolved through discussions with the translators.

The participants, or a caregiver or guardian for patients 15 years or less, were given oral and written information about the study and were informed that their participation was voluntary and that they would remain anonymous. If they agreed, walk-in patients, or their caregivers, completed the questionnaire while waiting for a consultation with the medical doctor. For children, their age, gender and immigrant status were recorded, along with the work and social welfare benefit status of their accompanying family member. The questionnaire took about 2 min to complete. Returning the completed questionnaire to the medical doctor at the end of the consultation was considered implied consent for study participation. Language barriers and illiteracy were overcome by using family members or health personnel as interpreters.

### Inclusion criteria

In our study we wanted to examine utilization of emergency care services among walk-in patients where attending a RGP could have been a relevant option. Patients of all ages except patients attending scheduled return visits were included. Patients arriving with severe urgency levels and reduced ability to cooperate were thus not eligible for inclusion. This applied for patients admitted by ambulance, those triaged as “red priority” or who were assumed to need help within a few minutes, or those who were seriously intoxicated or having an acute psychiatric episode.

### Study sample

**Fig. 1:**
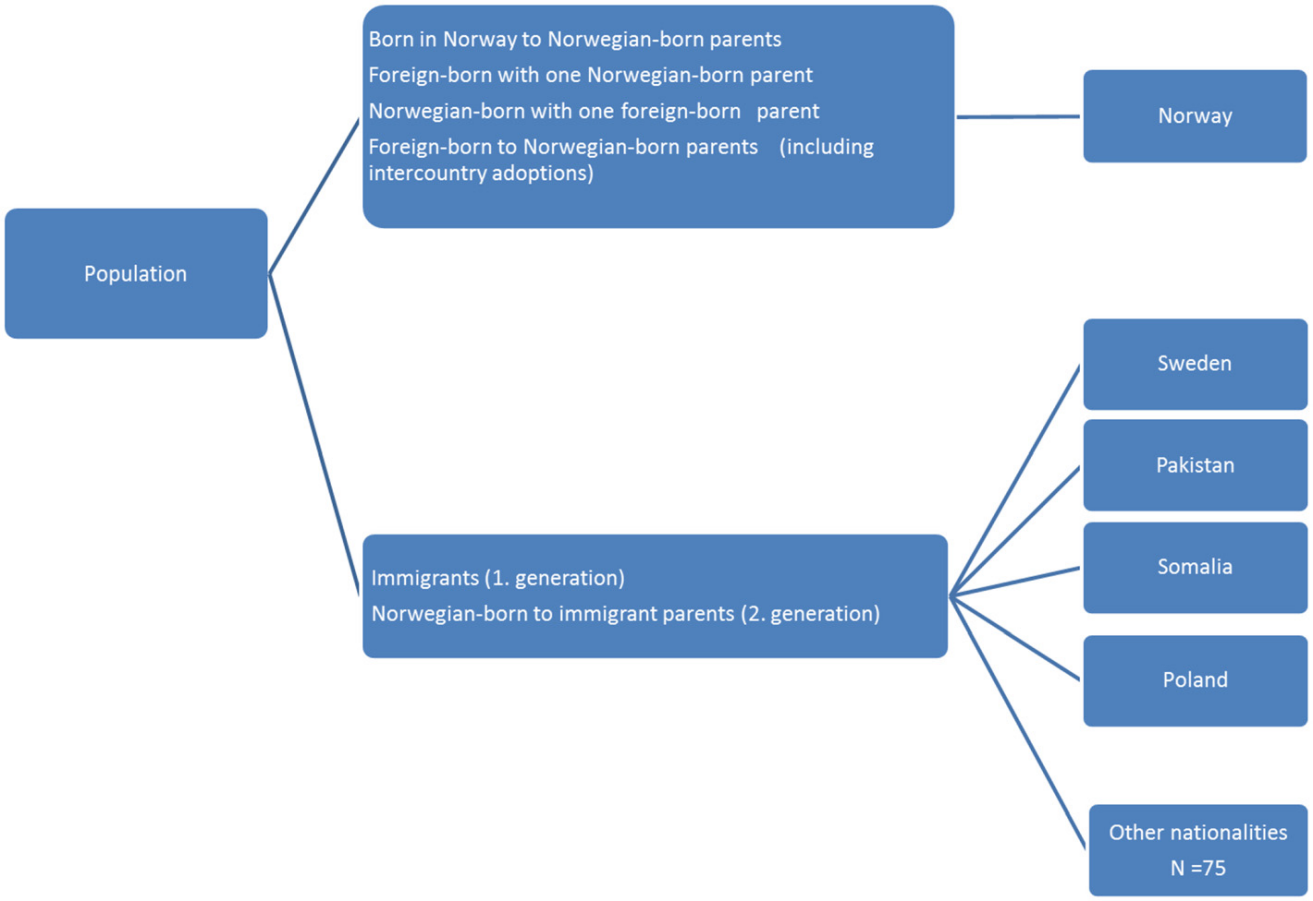
Classification of the patient population by immigration background. The country of origin is based on the patient’s country of birth, or their mother’s country of birth if the patient was born in Norway

Patients were categorized based on immigration status and country of origin, according to the criteria and definitions used by Statistics Norway [17]. Patients were defined as being of non-Norwegian origin if they and both their parents were born abroad or if they were born in Norway but both parents were born abroad. Patients were divided into groups based on their immigration status and country of origin according to their birth country, or their mother’s country of birth if the patient was born in Norway (Fig. 1). In the official national statistics, patients with another immigration status, such as foreign-born with one Norwegian parent, Norwegian-born with one foreign-born parent or foreign-born with two Norwegian-born parents (including international adoptees) are classified as “the rest of the population”. The participants in our study were grouped as Norwegians, immigrants (first-generation immigrants) and Norwegian-born persons with immigrant parents (second-generation immigrants). “Norwegian” was defined by the common term referring to native Norwegians and persons classified as “the rest of the population”. We were not allowed to record participants’ personal identification numbers because this information is restricted for privacy and ethical reasons. Therefore, we were unable to classify the proportions of illegal or undocumented immigrants and thus we included all immigrants, regardless of legal status, in one group. The four most frequently represented countries among immigrants and Norwegian-born participants with immigrant parents (Sweden, Pakistan, Somalia and Poland) were selected for further analysis.

### Measures

We analysed gender, age, immigration status, work status and country of origin. We also analysed self-reported utilization rates of OAEOC and RGP services during the preceding 12 months. The self-reported affiliation status with the RGP patient system was categorized as “yes”, “no” or “do not know”.

### Analyses

The questionnaires were coded and entered into a database using EpiData Software version 2.2. (EpiData Association) and analysed with SPSS version 22.0 and STATA version 13.3. Descriptive statistics, including proportions and means, were calculated. Pearson’s chi-square test was used to identify associations between categorical variables and one-way analysis of variance (ANOVA) was used to identify differences between means. Two different approaches were used to analyse OAEOC utilization patterns. In the first approach, we used Poisson regression analyses adjusted for age and gender to assess participants’ OAEOC and RGP visit frequencies. In the second approach, we used Pearson’s chi-square and Z-proportion tests to compare the proportions of first- and second-generation immigrants and those from the four most frequently represented countries among the patient population, with their respective proportions within the general Oslo population. For the gender- and age-stratified proportion analyses, we used bootstrapping to create 95 % confidence intervals (CIs). Significance was identified as the 5 % level (*p* < 0.05).

### Ethical approval

The study was voluntary and anonymous, so ethical approval was not required. However, the study was presented to the Norwegian Data Protection Authority, the Oslo University Hospital Information Security and Privacy Office, and the Regional Committees for Medical and Health Research Ethics in Norway and received no further comments or restrictions, given that no personal identification or diagnosis data were collected.

## Results

**Fig. 2:**
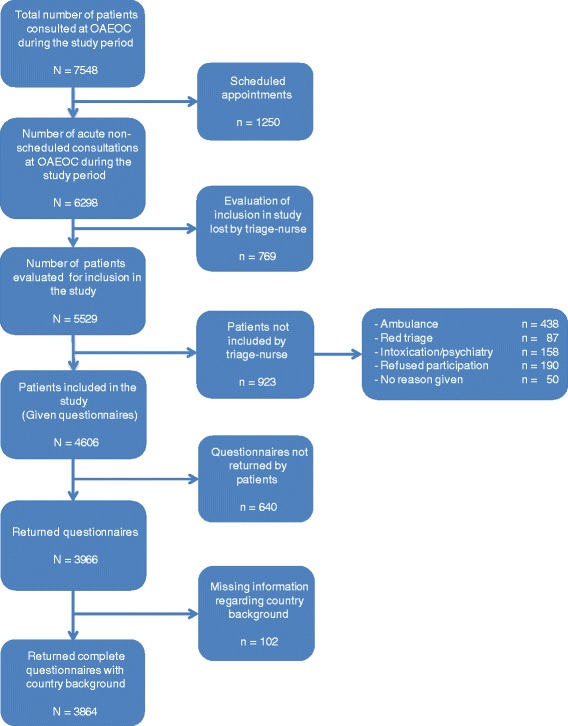
Flow chart of study participant inclusion

During the study period, 6298 emergency patients were seen at the OAEOC (Fig. 2). Among these, 769 (12 %) were not considered for inclusion for practical reasons such as urgency or time constraints at the emergency clinic. A total of 5529 patients were evaluated for participation by the triage nurse. Among these, 2753 were seen at the general emergency clinic and 2776 at the trauma clinic. Among those evaluated, 923 patients were not included because they were emergency admissions, they indicated that they did not want to participate, or they gave no reason for not participating. Of the 4606 walk-in patients given a questionnaire by the triage nurse, 3864 (response rate 84 %) returned a complete questionnaire with country background information (1821 from the general emergency clinic and 2043 from the trauma clinic). Immigrants represented 79 nationalities. Of the 1364 participants who had an immigration background, 79.2 % preferred the Norwegian language version of the questionnaire, 10.4 % the English version, 5.1 % Polish, 3.2 % Somali, 1.0 % Urdu, 0.7 % Farsi (Persian) and 0.4 % Sorani (Kurdish).

### Characteristics of the OAEOC study participants

A greater proportion of Norwegians utilized the trauma clinic compared to the general emergency clinic, while among first- and second-generation immigrants it was the opposite (Table 1). Within each immigrant group, males were significantly over-represented at the OAEOC, whereas no gender difference was observed in the pattern of OAEOC use by Norwegians. The mean age of the immigrant and Norwegian patients was 26.6 and 29.6 years, respectively. Second-generation immigrants where generally younger, with a mean age of 9.7 years and 86 % were under 20 years of age. The employment rate was 58.9 % for all immigrants and 61.3 % among Norwegians. First-generation immigrants were more likely to receive some form of social welfare benefits (14.8 %) compared with Norwegians (9.2 %). Patients reporting high use (≥3 visits) of the OAEOC during the preceding 12 months were higher in both first- and second-generation immigrants compared with Norwegians. They also had a higher mean number of visits. Among patients registered with the RGP scheme significantly more first-generation immigrants reported ≥3 visits with their RGP during the preceding 12 months than the Norwegians did. The proportion of patients who reported being registered with the RGP patient system was 75.1 % for all immigrants compared with 95.5 % for Norwegians. Registration rates differed between first- (71.0 %) and second-generation immigrants (95.7 %). The proportion of patients who did not know whether they were registered with an RGP was significantly higher among first-generation immigrants than among Norwegians.

**Table 1:** Characteristics of immigrant groups within the study population compared with Norwegians

	Norwegians	Immigrants		
		First generation	Second generation	Total^a^
Number of patients (%)				
OAEOC	2500 (100)	1004 (100)	360 (100)	1364 (100)
DEGP (general emergency clinic)	1053 (42.2)	576 (57.4)**	192 (53.3)**	768 (56.3)*
SOE (trauma clinic)	1447 (57.8)	428 (42.6)**	168 (46.7)**	596 (43.7)*
Gender (%)				
Female	1245 (50.1)	450 (45.3)*	133 (38.9)**	583 (43.7)**
Male	1241 (49.9)	543 (54.7)*	209 (61.1)**	752 (56.3)**
Age in years, mean (SD)	29.6 ± 20.9	32.6 ± 14.4**	9.7 ± 10.2**	26.6 ± 16.7**
Paediatric/adolescent proportion, 0–19 years (%)	812 (33.0)	104 (10.8)**	292 (85.6)**	396 (30.4)
Work status (%) ^b^				
Employed	1485 (61.3)	600 (63.3)	149 (46.1)**	749 (58.9)
Social welfare benefits	222 (9.2)	140 (14.8)**	27 (8.4)	167 (13.1)**
Other^c^	716 (29.6)	208 (21.9)**	147 (45.5)**	355 (27.9)
Self-reported use of OAEOC during the preceding 12 months (%)				
No visits	1355 (55.0)	465 (47.8)**	118 (34.5)**	583 (44.4)**
1–2 visits	828 (33.6)	366 (37.7)**	141 (41.2)**	507 (38.6)**
≥ 3 visits	279 (11.3)	141 (14.5)*	83 (24.3)**	224 (17.0)**
Mean number of visits	0.8 ± 1.2	1.1 ± 1.3**	1.5 ± 1.4**	1.2 ± 1.3**
Self-reported use of RGP during the preceding 12 months (%)^d^				
No visits	522 (22.7)	146 (21.6)	61 (18.9)	207 (20.7)
1–2 visits	997 (43.4)	222 (32.9)**	145 (44.9)	367 (36.8)**
≥ 3 visits	777 (33.8)	307 (45.5)**	117 (36.2)	424 (42.5)**
Mean number of visits	1.9 ± 1.4	2.2 ± 1.5**	2.0 ± 1.4	2.1 ± 1.5**
Self-reported RGP registration status (%)				
Yes	2326 (95.6)	689 (71.0)**	336 (95.7)	1025 (75.1)**
No	69 (2.8)	250 (25.7)**	8 (2.3)	258 (19.5)**
Do not know	37 (1.5)	32 (3.3)**	7 (2.0)	39 (3.0)*

OAEOC (Oslo Accident and Emergency Outpatient Clinic), Missing data: Gender (*n* = 43), Work status (*n* = 170), OAEOC visits (*n* = 88), RGP visits (*n* = 57), RGP status (*n* = 110)

*Indicates a significant difference compared with Norwegians (*p* < 0.05), **p < 0.001

^a^ Total immigrants (first generation) and Norwegian-born with immigrant parents (second generation)

^b^ Work status of the relatives accompanying patients < 16 years

^c^ Other: pensioner, student or homemaker

^d^ Includes only patients who report having an RGP (*n* = 3351)

### Characteristics of participants from four selected countries compared with Norwegians

Thirty-eight percent of first- and second-generation immigrants originated from Sweden, Pakistan, Somalia or Poland. The pattern of OAEOC use among immigrants from these four countries was compared with Norwegians (Table 2). In contrast to Norwegians, three of the four country-based immigrant groups made greater use of the general emergency clinic, compared with the trauma clinic. Gender differences did not reach statistical significance. Patients originating from Pakistan and Somalia were significantly younger compared with Norwegians. There was marked variance in the proportion of patients <20 years of age. Patients from Sweden and Poland had higher rates of employment compared with all groups, including Norwegians, in contrast to the Pakistan and Somalia groups among whom rates were significantly lower. The Somali group received social welfare benefits at significantly higher rates. Patients from Sweden, Pakistan and Somalia reported significantly more OAEOC visits during the preceding 12 months than the Norwegians did. Self reported use of RGPs differed between Norwegians and those from the four selected countries inasmuch as patients from Pakistan reported higher use whereas those from Sweden and Poland reported lower use. Compared with Norwegians, the proportion of those who reported being registered with the RGP system was lower for three of the four immigrant subgroups, those from Pakistan being the exception.

**Table 2:** Characteristics of the study population from selected countries compared with Norwegians

	Norway	Sweden	Pakistan	Somalia	Poland
Number of patients (%)					
OAEOC	2500 (100)	180 (100)	134 (100)	114 (100)	96 (100)
DEGP (general emergency clinic)	1053 (42.2)	110 (61.1)**	73 (54.5)*	69 (60.5)**	50 (52.1)
SOE (trauma clinic)	1447 (57.8)	70 (38.9)**	61 (45.5)*	45 (39.5)**	46 (47.9)
Gender (%)					
Female	1245 (50.1)	90 (50.0)	64 (48.5)	49 (44.5)	39 (40.6)
Male	1241 (49.9)	90 (50.0)	68 (51.5)	61 (55.5)	57 (59.4)
Age in years, mean (SD)	29.6 ± 20.9	25.9 ± 11.7*	25.3 ± 18.1*	18.7 ± 15.3*	29.1 ± 15.6
Paediatric/adolescent proportion, 0–19 years (%)	812 (33.0)	17 (9.5)**	53 (41.7)*	57 (56.4)**	18 (19.1)*
Work status (%) ^a^					
Employed	1485 (61.3)	152 (84.4)**	61 (47.7)*	39 (39.8)**	69 (75.0)*
Social welfare benefits	222 (9.2)	11 (6.1)	12 (9.4)	15 (15.3)*	11 (12.0)
Other^b^	716 (29.6)	17 (9.4)**	55 (43)*	44 (44.9)*	12 (13.0)**
Self-reported use of OAEOC during the preceding 12 months (%)					
No visits	1355 (55.0)	86 (48.3)	53 (40.5)*	37 (34.9)**	49 (52.7)
1–2 visits	828 (33.6)	62 (34.8)	51 (38.9)	45 (42.5)	33 (35.5)
≥ 3 visits	279 (11.3)	30 (16.9)*	27 (20.6)*	24 (22.6)**	11 (11.8)
Mean number of visits	0.8 ± 1.2	1.1 ± 1.3*	1.4 ± 1.4**	1.4 ± 1.3**	0.9 ± 1.1
Self-reported use of RGP during the preceding 12 months (%) ^c^					
No visits	522 (22.7)	18 (31.6)	14 (11.7)*	20 (20.6)	23 (37.7)*
1–2 visits	997 (43.4)	28 (49.1)	44 (36.7)	41 (42.3)	23 (37.7)
≥ 3 visits	777 (33.8)	11 (19.3)*	62 (51.7)**	36 (37.1)	15 (24.6)
Mean number of visits	1.9 ± 1.4	1.5 ± 1.3*	2.5 ± 1.4**	2.0 ± 1.4	1.5 ± 1.5*
Self-reported RGP registration status (%)					
Yes	2326 (95.6)	57 (31.8)**	125 (96.9)	98 (90.7)*	61 (64.9)**
No	69 (2.8)	114 (63.7)**	3 (2.3)	8 (7.4)*	25 (26.6)**
Do not know	37 (1.5)	8 (4.5)*	1 (0.8)	2 (1.9)	8 (8.5)**

OAEOC (Oslo Accident and Emergency Outpatient Clinic), Missing data: Gender (*n* = 20), Work status (*n* = 103), OAEOC visits (*n* = 54), RGP visits (*n* = 36), RGP status (*n* = 82)

*Indicates a significant difference compared with Norwegians (*p* < 0.05), **p < 0.001

^a^ Work status of the relatives accompanying patients < 16 years

^b^ Other: pensioner, student, homemaker

^c^ Includes only patients who report having an RGP (*n* = 2667)

### Frequency of visits to the OAEOC and RGP during the previous 12 months

The frequency of OAEOC and RGP use was analysed with Poisson regression models adjusted for age and gender (Table 3). Both first- and second-generation immigrants reported more OAEOC and RGP visits compared with Norwegians (*p* < 0.001). Females reported higher frequencies of use of both OAEOC and RGP compared with males. The number of RGP visits increased with age, while the frequency of OAEOC visits was highest among young patients. With the exception of patients from Poland, the other country-based immigrant groups visited the OAEOC more frequently during the preceding 12 months compared with Norwegians. However, compared to Norwegians, immigrants from both Poland and Sweden had fewer RGP visits whereas those from Pakistan had significantly more.

**Table 3:** Frequency of visits to the OAEOC and RGP during the previous 12 months. Incidence rate ratios of different models analysed with Poisson regression across immigrant groups and selected countries

	OAEOC visits		RGP visits	
	Model 1	Model 2	Model 1	Model 2
	IRR (95 % CI)	IRR (95 % CI)	IRR (95 % CI)	IRR (95 % CI)
Model for immigrants				
Norwegians (ref)	1	1	1	1
First-generation immigrants	1.29 (1.17–1.42)**	1.34 (1.21–1.49)**	1.16 (1.09–1.23)**	1.12 (1.05–1.19)**
Second-generation immigrants	1.81 (1.58–2.07)**	1.58 (1.36–1.84)**	1.09 (0.99–1.19)	1.34 (1.21–1.46)**
Gender (ref: Female)		1		1
Male		0.90 (0.82–0.98)*		0.79 (0.75–0.83)**
Age (ref: < 20 years)		1		1
20–39		0.79 (0.71–0.88)*		1.17 (1.10–1.26)**
40–59		0.74 (0.64–0.85)**		1.46 (1.35–1.58)**
≥ 60		0.60 (0.49–0.72)**		1.77 (1.62–1.94)**
Model for selected countries				
Norway (ref)	1	1	1	1
Sweden	1.28 (1.04–1.56)*	1.32 (1.07–1.63)*	0.79 (0.63–0.98)*	0.78 (0.63–0.98)*
Pakistan	1.68 (1.35–2.09)**	1.62 (1.29–2.02)**	1.34 (1.18–1.52)**	1.37 (1.21–1.54)**
Somalia	1.73 (1.36–2.20)**	1.55 (1.19–2.01)**	1.05 (0.90–1.23)	1.12 (0.95–1.33)
Poland	1.02 (0.76–1.37)	1.01 (0.75–1.37)	0.78 (0.63–0.98)*	0.80 (0.65–0.99)*
Gender (ref: Female)		1		1
Male		0.90 (0.82–1.00)		0.78 (0.74–0.83)**
Age (ref: < 20 years)		1		1
20–39		0.79 (0.70–0.89)**		1.20 (1.12–1.29)**
40–59		0.69 (0.58–0.80)**		1.40 (1.29–1.53)**
≥ 60		0.58 (0.47–0.72)**		1.76 (1.60–1.93)**

OAEOC (Oslo Accident and Emergency Clinic), RGP (regular general practitioner)

Norwegians used as the reference group. *IRR* incidence rate ratio

Model 1: Unadjusted, Model 2: Adjusted for age and gender

* Significant result at the *p* < 0.05 level, **p < 0.001

### Characteristics of patients seen at the two clinics

Table 4 indicates that a higher proportion of male patients attended the trauma clinic (59 %) compared with the general emergency clinic (45 %; *p* < 0.05). This relative over-representation of men at the trauma clinic applied uniformly to Norwegians and all immigrants except for those from Pakistan, and was highest among patients from Somalia (74 %), Poland (70 %) and Sweden (66 %). Females (55 %), with the exception of patients from Pakistan (46 %), were seen most frequently at the general emergency clinic, with the highest proportions among patients from Sweden (60 %) and Norway (58 %). There was no significant difference in mean age between patients at the two clinics: 28.0 years (±19.5) at the general emergency clinic and 29.0 years (± 19.7) at the trauma clinic.

**Table 4:** Characteristics of participants seen at the DEGP and SOE stratified by gender and mean age

	DEGP (general emergency clinic)			SOE (trauma clinic)		
	*N* = 1798			*N* = 2023		
	Female	Male	Mean age	Female	Male	Mean age
	*n* (%)	*n* (%)	±SD	*n* (%)	*n* (%)	±SD
Norwegians	609 (58.3)	435 (41.7)	29.1 ± 21.1	636 (44.1)**	806 (55.9)**	30.0 ± 20.8
Immigrants	386 (51.2)	368 (48.8)	26.5 ± 16.9	197 (33.9)**	384 (66.1)**	26.8 ± 16.6
First-generation	303 (53.2)	267 (46.8)	32.5 ± 14.0	147 (34.8)**	276 (65.2)**	32.7 ± 14.7
Second-generation	83 (45.1)	101 (54.9)	8.0 ± 9.8	50 (31.6)*	108 (68.4)*	11.6 ± 10.3*
Total number of participants	995 (55.3)	803 (44.7)	28.0 ± 19.5	833 (41.2)**	1190 (58.8)**	29.0 ± 19.7
Selected countries^a^						
Sweden	66 (60.0)	44 (40.0)	24.5 ± 7.9	24 (34.3)**	46 (65.7)**	28.3 ± 15.8*
Pakistan	33 (45.8)	39 (54.2)	27.4 ± 20.5	31 (51.7)	29 (48.3)	22.8 ± 14.5
Somalia	38 (56.7)	29 (43.3)	18.0 ± 15.6	11 (25.6)*	32 (74.4)*	19.9 ± 14.9
Poland	25 (50.0)	25 (50.0)	28.5 ± 17.2	14 (30.4)	32 (69.6)	21.3 ± 10.5

Missing data: Gender (DEGP *n* = 23), (SOE *n* = 20)

*Indicates a significant difference in gender distribution between the clinics (*p* < 0.05), ** *p* < 0.001

^a^ Including both immigrants and Norwegian-born with immigrant parents

### OAEOC utilization in relation to groups’ population representation in Oslo

**Fig. 3:**
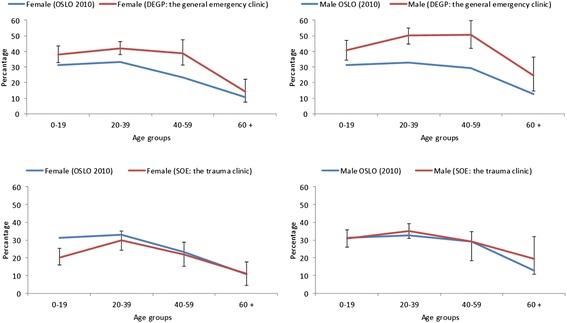
Distribution of patients with immigration background stratified by gender and age. The proportional representation (including both first- and second-generation immigrants) in the patient population at the general emergency clinic and the trauma clinic compared with the gender- and age-stratified proportions of this group in the Oslo population during 2010, according to Statistics Norway. Percentages and 95 % CIs are shown

Table 5 shows the unadjusted proportional representation of immigrant groups at the OAEOC, divided into first- and second-generation immigrants and by country of origin, in relation to their respective proportions of Oslo’s population. The representation of all immigrants (including first- and second-generation immigrants) seen at the OAEOC (35 %; *p* < 0.001) and the general emergency clinic (42 %; *p* < 0.001) was significantly higher compared with their proportion of Oslo’s population (27 %). When grouped by country of origin, those from Sweden, Somalia and Poland were most disproportionally represented at the OAEOC, compared with their proportion among the general city population. However, when immigrants who did not report having an RGP were excluded, only those from Somalia were still over-represented at both clinics (see Additional file 2). In addition, both first- and second-generation immigrants were still over-represented at the general emergency clinic. Figure 3 shows the distribution of patients with immigration background (first- and second-generation immigrants) who attended the general emergency clinic and the trauma clinic compared with their gender- and age-stratified proportions in the Oslo population according to Statistics Norway (for background data, see Additional file 3). Young and middle-aged females and males were significantly over-represented in the general emergency clinic patient population. Their representative proportions of the trauma clinic patient population were almost identical to those of the general population, except for a significant under-representation of young females (0–19 years). The age- and gender-adjusted proportional representations of patients from the selected countries are presented in an additional table (see Additional file 4). Swedish males and females, aged 20–39 years, were significantly over-represented in the patient population at both the general emergency clinic and the trauma clinic. Both male and female children and adolescents from Somalia (aged 0–19 years) were over-represented at the general emergency clinic while females were under-represented at the trauma clinic. The proportion of patients from Pakistan was equally distributed in the patient population at both clinics compared with their proportion in the Oslo population, except for Pakistani males aged 40–49 years, who were over-represented at the general emergency clinic. Polish males aged 20–39 years were over-represented at the trauma clinic while young and middle-aged Polish females, 0–39 years, were over-represented at the general emergency clinic compared with their predicted proportion of the general population.

**Table 5:** Proportional representation of patient groups compared with that in the general population of Oslo (2010)

	OSLO (ref)	OAEOC	DEGP	SOE
	% (*N* = 586,860)	% (*N* = 3864)	% (*n* = 1821)	% (*n* = 2043)
Norwegians	72.7	64.7**	57.8**	70.8
Immigrants	27.3	35.3**	42.2**	29.2
First generation	20.9	26.0**	31.7**	21.0
Second generation	6.5	9.3**	10.5**	8.2*
Selected countries^a^				
Sweden	1.8	4.7**	6.1**	3.5**
Pakistan	3.6	3.5	4.1	3.0
Somalia	1.3	3.0**	3.8**	2.3*
Poland	1.5	2.5**	2.8**	2.3*

OAEOC (Oslo Accident and Emergency Clinic), DEGP (general emergency clinic), SOE (trauma clinic)

*Indicates a significant difference compared with their proportion in the general population of Oslo (*p* < 0.05), ** *p* < 0.001

^a^ Including both first- and second-generation immigrants

## Discussion

### Study findings

Our data indicate that immigrants in Oslo, including both first-generation and second-generation immigrants, use the city’s walk-in emergency services more often than would be predicted by their representation within the general population. This conclusion is supported by the patients’ self-reported use of the emergency facilities during the previous 12 months. Utilization was higher at the general emergency clinic, whereas the proportion of immigrants at the trauma clinic was similar to the group’s representation in the general population of Oslo. Males were more frequently patients at the trauma clinic and females at the general emergency clinic. The OAEOC patient sample was generally younger than the general population. Approximately one-third of the patients were <20 years old. Of interest are also the different affiliation rates with the RGP scheme. First-generation immigrants reported a lower rate of registration with the RGP scheme than Norwegians, while second-generation immigrants’ rates were similar to those of Norwegians.

The second-generation immigrants living in Norway are mostly descendants of immigrants who arrived during the last decades and represent a relatively young population (mean age 9.7 years). They are generally integrated into the Norwegian health care system by having taken part in the obligatory Norwegian maternity and child health care services. Immigrants from Sweden and Poland, mainly labour immigrants, reported the lowest affiliation rates with the RGP scheme. Increased immigration, particularly by labour immigrants, entails that new perspectives are needed on how to organize the health care service to ensure access equity.

Increased utilization of emergency services by immigrants may reflect cultural differences in health literacy, knowledge about the health care system, difficulties in accessing an RGP and language barriers [3–6]. If immigrants walk into the emergency clinic instead of using the telephone to arrange an appointment with their RGP, this may explain their higher utilization of the OAEOC.

The four immigrant nationalities specifically examined in this study have some distinct features. Patients from Sweden and Poland are mostly labour immigrants. The group from Pakistan has predominantly immigrated to Norway since the late 1960s and in recent years for the purpose of reunion with their families. Somalis have come to Norway seeking protection as asylum seekers or refugees since the late 1990s. One major difference between these nationalities is the rate at which they are employed. The labour immigrants from Sweden and Poland have high employment rates, whereas the immigrants from Pakistan and Somalia report low employment rates. In general, labour immigrants come to Norway on short-term work permits and many are not eligible to register with the RGP scheme [15]. Workers at temporary staff recruitment agencies on short-term contracts do not qualify for registration with the Norwegian National Population Register. This may explain the low self-reported RGP affiliation rates among labour immigrants and may contribute to higher workloads in emergency health care clinics.

After adjusting the analysis to include only patients reporting an affiliation with the RGP system, we found that the proportions of patients from Sweden and Poland who attended the OAEOC were similar to their representations in the general Oslo population. The lack of an RGP registration among labour immigrants is thus an important contributing factor to increased workload for the OAEOC. Undocumented and illegal immigrants are not allowed to register with an RGP and this group of patients contributes to the low self-reported RGP affiliation among first-generation immigrants. Although there are no official statistics on the proportion of the total immigrant population that undocumented and illegal immigrants represent, estimates in 2009 indicated a population of 12,000–18,000 throughout Norway and we can assume that many live in Oslo [18].

Besides attending the OAEOC, undocumented and illegal immigrants have few public alternatives for receiving acute health care. Only one daytime GP office sees patients who are not registered with a RGP. Charity organizations are open two afternoons and evenings per week (a total of 7 h per week). Apart from this, undocumented and illegal immigrant patients must attend the OAEOC or one of the few, expensive private health care clinics in Oslo. These private clinics treat predominantly socio-economically advantaged individuals and those with private health insurance. Doctors at private clinics do not receive financial compensation from the Norwegian Health Economics Administration and there are no official statistics on how many private clinics exist or how many patients they treat.

Immigrants were over-represented at the general emergency clinic and reported higher utilization of both the OAEOC and their RGPs than did the Norwegian population, which may reflect poorer general health, negative evaluation of their own health status or different cultural understandings of health and illness [19–21]. A meta-analysis reported substantial evidence for the harmful health effects of perceived prejudice and discrimination (referred to as “minority stress”) across a range of mental health and physical health outcomes including depression, psychological distress, anxiety, hypertension and potential risk factors for disease such as obesity and substance abuse [22]. These factors may all lead to different health-seeking behaviours. In the eastern area of Oslo, where up to 40 % of the population belongs to minority ethnic groups, the life expectancy is 10 years lower than for those living in the western area of the city [23]. Studies in Norway have also reported increased morbidity among immigrants including cardio-vascular disease, diabetes mellitus and mental health problems, indicating a greater disease burden, which may explain part of the increased utilization of emergency care services [24–26].

Males from Poland and Sweden are often engaged in manual labour and are therefore exposed to more work-related injuries and accidents, possibly explaining their over-representation at the trauma clinic [27, 28]. In addition, males are generally more involved in violence and crime [29]. Studies have also shown that immigrant women of non-Western origins are less physically active and have lower levels of engagement in sports activities, which may explain their under-representation at the trauma clinic [30, 31].

### Comparison with previous research

Our finding of a proportional increase in the utilization of emergency health care services among immigrants is consistent with reports from several other countries [5, 9–12]. In contrast, a 2010 review of the European literature by Norredam et al. of emergency room utilization among immigrants compared with non-immigrants showed varying degrees of higher, equal and lower utilization [13].

Our results differ slightly from those obtained using a registry-based study of immigrants’ use of emergency primary health care in Norway during 2008 [6], which concluded that immigrants generally used emergency services less than did native Norwegians, although they also found substantial variation between immigrant groups. In their study, immigrant workers from Germany and Poland used emergency care considerably less frequently than did native Norwegians, whereas asylum seekers from Somalia and Iraq used these services more often. One likely explanation for the discrepancy between our studies is that the first study covered all of Norway, with many different forms of emergency primary care services, while ours focused on these services in a single, uniform facility in Oslo.

A study conducted by Statistics Norway during 2005–2006 based on self-reported visits found that the mean number of emergency primary health care consultations per year was 0.6 among the immigrant population compared with 0.4 among Norwegians [32]; in the present study, the self-reported numbers of visits were respectively 1.2 and 0.8. These numbers are higher than those reported by a Norwegian registry-based study, which found a mean of 0.17 visits to emergency primary health care by Norwegians and 0.11 and 0.21 visits by immigrants from high- and low-income countries, respectively [33]. This registry-based survey reported that a significantly lower proportion of immigrants used their GP compared with Norwegians. However, during the daytime, immigrants were more likely to be frequent GP users (> 7 visits) compared with native Norwegians, although there were differences between immigrant groups [34]. Older immigrants, labour immigrants and immigrants from high-income countries used GPs less often, whereas refugees and immigrants from middle-income countries were over-represented among frequent attenders. We found that labour immigrants with a low rate of registration with the RGP system were over-represented at the OAEOC compared with their representation within the population, which agrees with the findings of other studies [4, 10].

### Strengths and limitations of our study

This study was based on patients’ self-reports on a 24-h basis over 2 weeks in September 2009. This period was representative of a normal work schedule for both the general emergency clinic and the trauma clinic insofar as there were no medical epidemics and not many tourists during this time. We consider the 2-week sampling period sufficient to generate a representative sample of the patient population because there were a large number of visits during this period. Nevertheless, the relatively short observation period may have created a risk of sampling bias. In contrast to registry-based studies that require personal identification numbers, our individual survey approach included patients who were not registered in the Norwegian National Population Register, such as undocumented immigrants, rejected asylum seekers and labour immigrants on a short-term stay in Norway. Although we were unable to either identify or analyse this group separately, we consider this approach a strength of our study. Because there are no official registers for undocumented or illegal immigrants, we do not know the numbers or percentages of the patient population that they comprised. Asking the patients their status in a questionnaire such as ours would probably not be reliable since illegal respondents would be naturally reluctant to report their status.

The response rate of distributed questionnaires was 84 % and relative high compared with similar studies [3, 10, 35]. However, 769 patients were not considered for inclusion by the triage nurse due to the periodic extreme hectic times at the emergency clinic. To our knowledge, these patients lost for evaluation of inclusion were predominantly acutely ill and brought in by ambulance, police or outreach teams and would not have qualified for inclusion anyway. Given that the main purpose of the study was to explore the utilization of emergency clinics by walk-in patients, it is unlikely that these missing patients unduly affected the overall results.

This study had several limitations. First, it did not cover the entire patient population that utilized the emergency services but focused only on walk-in patients with non-urgent or semi-urgent health conditions for which attending an RGP would have been a reasonable option. For this reason, the data may be relevant only to the health care utilization of walk-in patients. Second, since the study covered only walk-in patients, we have no information about the immigration status of those excluded. It would have been relevant to explore how immigrants were represented in the categories of patients admitted to the OAEOC by ambulance and emergency outreach teams, or their representation among those experiencing intoxication or psychiatric episodes. Third, we have no information about emergency health care utilization among people not using the OAEOC. Assuming that some are frequent visitors to the OAEOC while others rarely use the facility, the results may be relevant only for exploring the utilization patterns among the patient population at the emergency outpatient clinic. Recall bias may have affected patients’ self-reported patterns of utilization of both emergency services and RGPs. Over-reporting may also be more common in immigrants [6].

### Alternative explanations

Based on our survey analyses, we conclude that immigrants are over-represented at the general emergency clinic because of their high proportion among the emergency patient population compared with their representation within the general Oslo population. Alternatively, it can be argued that this apparent over-representation reflects under-representation of Norwegians at the OAEOC due to their use of private emergency health clinics. Our impression from general practice in Oslo is that this is not the case, but this alternative hypothesis is difficult to investigate scientifically due to lack of epidemiological data from the private clinics.

### Relevance of the findings and recommendations for further research

Our findings have implications for the organization of the primary health care system for immigrants who come to Oslo on work permits. Initiatives that encourage immigrants to use RGPs for their regular health care needs could relieve some of the pressure on the city’s emergency health care services. However, it is difficult for immigrants on short-term work permits to join the RGP scheme. Providing accessible RGP services to immigrants who come to Norway on short-term visits may improve primary health care services for these patients.

Another unresolved issue is the higher utilization of health care services among immigrants in general and among specific groups. Further research is needed to understand the issues related to health disparities or culturally dependent differences in health-seeking behaviour.

## Conclusions

In Oslo, immigrant subgroups use emergency health care services differently. Increased use was seen mostly at the general emergency clinic, whereas the proportion of immigrants at the trauma clinic was similar to the general population. Labour immigrants from Sweden and Poland used emergency health care services more frequently than Norwegians did, and had low registration rates in the RGP system. Immigrants overall reported higher rates of utilization of both emergency health care services and RGPs. These different patterns of health-seeking behaviour are important when planning and designing emergency and primary health care services for immigrants in large cities such as Oslo.

## Additional files

Additional file 1:
**English version of the questionnaire used in the study.** The questionnaire has three parts: I. Request and information for participation in the study; II. 15-item questionnaire completed by patients; and III. 7-item registration form for medical doctors. Part III is in Norwegian. (PDF 209 kb) Additional file 2:
**Proportional representation of patient groups compared with that in the general population of Oslo when patients who did not report an RGP assignment are excluded.** The unadjusted proportional representation of immigrant groups at the OAEOC, divided into first- and second-generation immigrants and by country of origin, in relation to their respective proportions within the Oslo population. (PDF 172 kb) Additional file 3:
**Proportional representation of patient groups compared with their proportion in the general population of Oslo.** The proportional representation (including both first- and second-generation immigrants) in the patient population at the general emergency clinic and the trauma clinic compared with the gender- and age-stratified proportions of this group in the population of Oslo. (PDF 178 kb) Additional file 4:
**Proportional representation of patient groups based on selected countries compared with their proportion in the general population of Oslo.** The proportional representation (including patients from Sweden, Pakistan, Somalia and Poland) in the patient population at the general emergency clinic and the trauma clinic compared with the gender- and age-stratified proportions of this group in the population of Oslo. (PDF 179 kb)

## Abbreviations

CI: Confidence interval
DEGP: Department of Emergency General Practice (general emergency clinic)
IRR: Incidence rate ratio
OAEOC: Oslo Accident and Emergency Outpatient Clinic
RGP: Regular general practitioner
SD: Standard deviation
SOE: Section for Orthopaedic Emergency (trauma clinic)
