# Supplementary material for: Use of emergency care services by immigrants—a survey of walk-in patients who attended the Oslo Accident and Emergency Outpatient Clinic
Source: BMC Emerg Med. 2015 Oct 7;15:25. doi: 10.1186/s12873-015-0055-0 (PMC4596368; doi:10.1186/s12873-015-0055-0)
Supplement: Additional file 1: — English version of the questionnaire used in the study. The questionnaire has three parts: I. Request and information for participation in the study; II. 15-item questionnaire completed by patients; and III. 7-item registration form for medical doctors. Part III is in Norwegian. (PDF 209 kb) [file 12873_2015_55_MOESM1_ESM.pdf]

UNIVERSITETET I OSLO HELSE SØR-ØST Oslo kommune

[engelsk]

Serial number: …………
(For keeping the papers in order. Cannot be used to identify you.)

Request for your participation in the project

"EQUAL HEALTH SERVICES"

We who are responsible for the emergency service in metropolitan Oslo want to know more about how the various patient groups use the emergency service (*legevakten*), and how the emergency service is used in relation to the regular GPs. We are especially interested in how immigrants feel about the emergency service after they have used it.

To get as complete a picture as possible, we are now asking that ***all*** patients (with the exception of those who are acutely ill) answer some questions on why they call/go to the emergency service.

You are hereby invited to participate in this study.

Voluntary participation
Participation is voluntary. If you do not want to take part, you do not need to give a reason, and this will have no consequences for the treatment you receive from the emergency service.

All the information you give will be registered anonymously so that nothing can be traced back to you after you have answered the questionnaire. It will not be possible to use the project results to identify you when they are published.

What does the project mean for you?
If you are willing to take part in the survey, while you are waiting to be seen by a doctor, we will ask you to answer some questions on the next pages as best you can.

If you are with a sick child or a sick adult, we ask that you answer the questions from the patient's perspective (experiences). Give the completed questionnaire to the doctor when the consultation is over. The doctor will fill in some information on how the consultation has gone and will then give the anonymous form to the project management team. The University of Oslo will process the forms and analyse the data.

Possible advantages and disadvantages
Your participation in the project will not give you any special advantages, but your answers will help us plan future activities at the emergency service so that we will be better able to address the various needs that patients have. You will be asked to fill in the questionnaire while you are waiting to see the doctor, so filling it in will not prolong your wait.

Who is in charge of the survey?
This is a joint project involving Helse Sør-Øst RHF (South-Eastern Norway Regional Health Authority), Oslo local authority and the University of Oslo, in cooperation with Allmennlegevakten Oslo kommune (General Emergency Service, City of Oslo), Skadelegevakten Oslo (Injury Emergency Service Oslo), Ullevål University Hospital, Allmennlegevakten Skedsmo kommune (General Emergency Service, Skedsmo), Skadelegevakten Lillestrøm Ahus (Injury Emergency Service, Lillestrøm Ahus), and Akuttmottaket Ahus (Emergency Service Ahus).

The South-Eastern Norway Regional Health Authority has the formal responsibility for the project.

You who are filling in this form are a:

☐ patient ☐ relative/family member of a child (patient)

☐ relative/family member of an adult patient ☐ other: ………………………….…

Is the patient: ☐ Female ☐ Male

How old is the patient: ……..… years old

Where does the patient live: ☐ Oslo ☐ Akershus

☐ Outside Oslo/Akershus ☐ No fixed address

What country was the patient born in: ……………………..………...

What country was the patient's mother born in: ………………………..……………………….............

What country was the patient's father born in: …………………...

Native language (the language you speak within the family): …………………………………...………....

Does the patient have a regular GP: ☐ Yes (perhaps same as mother/father) ☐ No ☐ Don't know

In what county does the patient have his/her regular GP: ☐ Oslo ☐ Akershus

☐ Other county ☐ Do not have reg. GP

How many times has the patient had an appointment with the regular GP the last 12 months:

0	1	2	3	4 or more
☐	☐	☐	☐	☐

How many times (not counting this time) has the patient been to the emergency service (*legevakten*) the last 12 months:

0	1	2	3	4 or more
☐	☐	☐	☐	☐

Considering the health problems that have brought you/ the patient to the emergency service today, how urgent is it, in your opinion, to be examined by a doctor?

☐ Very urgent. I (the patient) must have help within an hour or sooner

☐ Fairly urgent. I (the patient) must have help within a few hours

☐ Not so urgent. I (the patient) could perhaps have waited until tomorrow

What is your current status? (Applies for the patient. If the patient is a child, then this applies to the person who is accompanying the child) Tick the box with the alternative that fits your status best.

☐ Working

☐ On sick leave/disability benefit/rehabilitation

☐ Pensioner

☐ Attending education, pupil/student

☐ Working at home

☐ Unemployed

☐ Other: what ……………………………………………………………………………

Why did you choose the emergency service instead of your regular GP today:

(Choose one of the three boxes A, B or C below)

A

☐ **I/we have tried to contact the regular GP today, but:**

***Reason** (Tick **the most** important. Tick only **one** box):*

- The regular GP office was closed ☐
- I/we could not get through on the phone ☐
- I/we could not book an appointment soon enough ☐
- The regular GP office asked me/us to use the emergency service ☐
- Other; describe in the last box at the bottom of the page ☐

or

B

☐ **I/we have NOT tried to contact the regular GP today, because:**

***Reasons** (tick **up to three** of the most important:*

- I/the patient have/has a regular GP in another district/municipality ☐
- I/the patient became ill outside normal working hours ☐
- It is difficult getting to the regular GP in the daytime ☐
- Bad experience from previous attempts at contacting the regular GP ☐
- It is quicker to get help from the emergency service ☐
- I/we do not feel the regular GP provides the help we need now ☐
- I want to decide myself when to go to the doctor ☐
- I called the emergency service switchboard, they told me to come here ☐
- I/the patient do/does not have a regular GP ☐
- Other; describe in the last box at the bottom of the page ☐

or

C

☐ **I/the patient have/has an acute injury. The emergency service is the best place where we can get help for this. I/we have therefore come to the emergency service directly without contacting other doctors.**

☐ **Other (write in this box if there are other reasons why you have preferred the emergency service to your regular GP. But don't write anything that can identify you):**

....

....

....

Thank you for answering the questions and helping to make the emergency service better.
Please give this form to the doctor you are going to see.

This page is to be filled in by the doctor. The questions are about how serious the doctor feels your illness is, how you/the patient should be followed up after your visit to the emergency service and if there were language or other cultural challenges during the consultation.

Fylles ut av behandlende lege etter endt konsultasjonen

Dato: ………………..…   ☐ Dagtid (08-15)   ☐ Kveld (15-23)   ☐ Natt (23-08)

Din vurdering av alvorlighetsgrad av sykdom/skade ved denne henvendelsen på legevakt:

Meget alvorlig (behov for hjelp innen en time) ☐

Alvorlig (behov for hjelp innen få timer) ☐

Mindre alvorlig (kunne ha ventet til neste dag for vurdering hos fastlege) ☐

eller

Akutt skade (gjelder bare ved skadelegevaktene) ☐

Tiltak ved konsultasjonens slutt:

Pasienten ferdigbehandlet på legevakt ☐

Kontroll/operasjon på legevakten ☐

Videre oppfølging/vurdering hos fastlege ☐

Innleggelse/henvisning til akutt vurdering på sykehus ☐

Henvisning til sykehus poliklinikk/spesialist (elektiv eller påfølgende dag) ☐

Innlagt observasjonsposten (gjelder kun legevakten i Oslo) ☐

Annet: ……………………………………………………………………………………..

**Opplevde du språkvansker under konsultasjonen?**	Ikke relevant	Ikke i det hele tatt	I liten grad	I noen grad	I stor grad	I svært stor grad
	☐	☐	☐	☐	☐	☐

Hvis språkvansker, hvordan ble dette løst?

Profesjonell tolk ☐

Telefontolk ☐

Familiemedlem $<$ 16 år tolket ☐

Familiemedlem $\geq$ 16 år tolket ☐

Venn/kollega/pårørende av pasienten tolket ☐

Arbeidskollega tolket ☐

Snakket selv et utenlandsk språk med pasienten ☐ hvilket? …………………………..

Annet: ……………………………………….……………………………………………..

Utfordringer under konsultasjonen som du mener er av kulturell art:
(skriv ikke noe som kan identifisere den aktuelle pasienten)

……………………………………………………………………………………………...

……………………………………………………………………………………………
