# Supplementary material for: Use of emergency care services by immigrants—a survey of walk-in patients who attended the Oslo Accident and Emergency Outpatient Clinic
Source: BMC Emerg Med. 2015 Oct 7;15:25. doi: 10.1186/s12873-015-0055-0 (PMC4596368; doi:10.1186/s12873-015-0055-0)
Supplement: Additional file 2: — Proportional representation of patient groups compared with that in the general population of Oslo when patients who did not report an RGP assignment are excluded. The unadjusted proportional representation of immigrant groups at the OAEOC, divided into first- and second-generation immigrants and by country of origin, in relation to their respective proportions within the Oslo population. (PDF 172 kb) [file 12873_2015_55_MOESM2_ESM.pdf]

	OSLO (ref)	OAEOC	DEGP	SOE
	% (N = 586860)	% (N = 3351)	% (n = 1586)	% (n = 1765)
Norwegians	(72.7)	(69.4)**	(63.6)**	(74.7)
Immigrants and Norwegian-born with immigrant parents	(27.3)	(30.6)**	(36.4)**	(25.3)
Immigrants (1.generation)	(20.9)	(20.6)	(25.2)**	(16.4)**
Norwegian-born with immigrant parents (2. generation)	(6.5)	(10.0)**	(11.2)**	(9.0)**
Selected countries[1]				
Sweden	(1.8)	(1.7)	(1.8)	(1.6)
Pakistan	(3.6)	(3.7)	(4.3)	(3.2)
Somalia	(1.3)	(2.9)**	(3.8)**	(2.2)**
Poland	(1.5)	(1.8)	(1.9)	(1.8)

OAEOC (Oslo Accident and Emergency Outpatient Clinic), DEGP (general emergency clinic), SOE (trauma clinic)

[1]Including both immigrants and Norwegian-born with immigrant parents.

*Indicates a significant difference ($p < 0.05$), ** $p < 0.001$
