# Supplementary material for: Use of emergency care services by immigrants—a survey of walk-in patients who attended the Oslo Accident and Emergency Outpatient Clinic
Source: BMC Emerg Med. 2015 Oct 7;15:25. doi: 10.1186/s12873-015-0055-0 (PMC4596368; doi:10.1186/s12873-015-0055-0)
Supplement: Additional file 3: — Proportional representation of patient groups compared with their proportion in the general population of Oslo. The proportional representation (including both first- and second-generation immigrants) in the patient population at the general emergency clinic and the trauma clinic compared with the gender- and age-stratified proportions of this group in the population of Oslo. (PDF 178 kb) [file 12873_2015_55_MOESM3_ESM.pdf]

	Males				Females			
	OAEOC %(N=1941)	DEGP %(n=779)	SOE %(n=1162)	OSLO (ref) %(N=290013)	OAEOC %(N=1790)	DEGP %(n=974)	SOE %(n=816)	OSLO (ref) %(N=296847)
**Age: (0 – 19) years**								
Norwegians	65.4*	59.2**	69.2	68.6	71.0**	62.0*	79.8**	68.7
Immigrants	34.6*	40.8**	30.8	31.3	28.9	38.0*	20.2**	31.3
First-generation	7.7	6.9	8.2	7.6	9.5	12.5*	6.6	7.7
Second-generation	26.9*	33.9**	22.6	23.7	19.4*	25.5	13.6**	23.6
**Age (20 – 39) years**								
Norwegians	58.6**	49.6**	65.0	67.3	62.7*	58.1**	70.3	66.9
Immigrants	41.4**	50.4**	35.0	32.7	37.3*	42.0**	29.7	33.1
First-generation	38.3**	46.3**	32.7	29.3	34.9**	40.0**	26.7	29.8
Second-generation	3.1	4.1	2.3	3.4	2.4	2.0	3.0	3.3
**Age (40 – 59) years**								
Norwegians	62.5**	49.3**	70.9	70.7	70.1*	61.1**	78.0	76.5
Immigrants	37.5**	50.7**	29.1	29.3	29.9*	38.9**	22.0	23.4
First-generation	37.2**	50.7**	28.6	29.1	29.9*	38.9**	22.0	23.2
Second-generation	0.3	0	0.5	0.2	0	0	0	0.2
**Age (60 +) years**								
Norwegians	78.2**	75.4*	80.6*	87.3	87.5	85.7	89.0	89.4
Immigrants	21.8**	24.6*	19.4*	12.7	11.5	14.3*	11.0	10.7
First-generation	21.8**	24.6*	19.4*	12.6	11.4	13.1	9.9	10.6
Second-generation	0	0	0	0.1	0.1	1.2	1.1	0.1

Missing data (n = 133)

OAEOC: Oslo Accident and Emergency Outpatient Clinic, DEGP: the general emergency clinic, SOE: the trauma clinic
