# Supplementary material for: Use of emergency care services by immigrants—a survey of walk-in patients who attended the Oslo Accident and Emergency Outpatient Clinic
Source: BMC Emerg Med. 2015 Oct 7;15:25. doi: 10.1186/s12873-015-0055-0 (PMC4596368; doi:10.1186/s12873-015-0055-0)
Supplement: Additional file 4: — Proportional representation of patient groups based on selected countries compared with their proportion in the general population of Oslo. The proportional representation (including patients from Sweden, Pakistan, Somalia and Poland) in the patient population at the general emergency clinic and the trauma clinic compared with the gender- and age-stratified proportions of this group in the population of Oslo. (PDF 179 kb) [file 12873_2015_55_MOESM4_ESM.pdf]

	Males				Females			
	OAEOC %(N=1941)	DEGP %(n=779)	SOE %(n=1162)	OSLO (ref) %(N=290013)	OAEOC %(N=1790)	DEGP %(n=974)	SOE %(n=816)	OSLO (ref) %(N=296847)
**Age: (0 – 19) years**								
Norway	65.4*	59.2*	69.2	68.6	71.0	62.0*	79.8**	68.7
Sweden	1.2*	0.4	1.7*	0.5	1.7**	2.7**	0.7	0.6
Pakistan	4.3	5.3	3.7	5.5	4.7	4.9	4.4	5.6
Somalia	4.6	6.5*	3.5	4.2	4.3	6.8*	1.8*	4.2
Poland	1.1	1.2	1.0	0.9	2.1**	3.0**	1.1	0.8
Other countries[1]	23.4*	27.4*	20.9	20.3	16.2	20.6	12.2**	20.1
**Age: (20 – 39) years**								
Norway	58.6**	49.6**	65.0	67.3	62.7*	58.1**	70.3	66.9
Sweden	9.0**	12.2**	6.7*	3.1	9.4**	11.5**	5.9*	3.2
Pakistan	2.5*	3.6	1.7*	3.9	2.9	2.2*	4.0	4.0
Somalia	2.1	2.4	1.9	2.0	1.8	2.0	1.3	1.9
Poland	4.4*	4.2	4.6*	3.2	2.4	3.8**	2.0	1.7
Other countries[1]	23.4*	28.0**	20.1	20.5	20.8	22.4	16.5*	22.3
**Age: (40– 59) years**								
Norway	62.5**	49.3**	70.9	70.7	70.1*	61.1**	78.0	76.5
Sweden	2.3	1.5	2.8*	1.4	0.4	0.8	0	1.3
Pakistan	1.9	5.9*	1.9	2.9	3.6	4.6	2.7	2.9
Somalia	2.5	2.9	2.3	1.5	1.4	3.1*	0	0.9
Poland	3.4	4.4	2.8	2.6	3.2**	2.3	3.3*	1.0
Other countries[1]	27.4*	36.0**	19.3	20.9	21.3	28.1**	16.0	17.4
**Age: (60 +) years**								
Norway	78.2*	75.4*	80.6*	87.3	86.3	85.7**	89.0	89.4
Sweden	0	0	0	0.7	2.9*	1.2	4.4**	1.0
Pakistan	3.8	6.6	1.4	2.0	1.1	2.4	0	1.0
Somalia	0	0	0	0.2	0	0	0	0.2
Poland	0	0	0	0.3	0.6	1.2	0	0.3
Other countries[1]	18.0*	18.0*	18.0*	9.5	9.2	9.5	6.6	8.1

Missing data: n = 133

OAEOC: Oslo Accident and Emergency Outpatient Clinic, DEGP: the general emergency clinic, SOE: the trauma clinic

[1]Pooled proportion of the remaning 75 countries
